# Assessing the impact of sulfur cap on bunkering spot selection in the ARA region

**DOI:** 10.1007/s13437-021-00257-9

**Published:** 2022-03-14

**Authors:** Orestis Schinas, Georgios Ourolidis

**Affiliations:** Department of Maritime & Logistics, Hamburg School of Business Administration, Willy-Brandt-Straße 75, 20459 Hamburg, Germany

**Keywords:** IMO2020, Sulfur cap, Bunkering port, Maritime logistics, Port competitiveness, Fuzzy TOPSIS, KPF

## Abstract

The introduction of IMO2020, the outbreak of COVID-19, and the oil price drop in 2020 had a significant impact on operators’ operating profits. Therefore, a competitive analysis of bunkering spots that suggests the optimal location for bunkering is of interest. This paper uses a combination of primary and secondary research, both from operators’ and suppliers’ side, to identify the key performance factors (KPFs) affecting the decision-making process of a bunkering port selection process. Answers were then combined by using a fuzzy TOPSIS analytical approach to quantify the competitive position of each port in the Amsterdam-Rotterdam-Antwerp (ARA) region. Results suggest that availability of low sulfur bunkers, bunker quality, bunker price, reliability, punctuality, and safety of bunkering services, as well as usage and availability of barges are the key KPFs in order of importance. Sulfur cap has not changed the competitive environment in the region as the geographic advantage of the port of Rotterdam plays a crucial role in the comparison with the other ports, in contrast to expressed concerns in the industry.

## Introduction

Bunkering port selection is a multidimensional problem with many conflicting parameters and therefore a multi-criteria decision-making model is required. The need for such a model is reinforced with the introduction of IMO2020 sulfur cap, that coincided with the recent COVID-19 epidemic outbreak, that merely resulted in the unprecedent low levels of oil prices, that consequently caused a potential displacement of the current bunkering port status quo as they affect both the demand and the supply side (quality and price). Following these foundations, this paper explores the factors affecting bunkering competitiveness at the ports of Northern Europe, specifically at the ports of Amsterdam, Rotterdam, and Antwerp.

These specific ports were selected because of their importance in the European trade and tourism. It is estimated that the Amsterdam-Rotterdam-Antwerp (ARA) region gathers approximately 50,000 ship calls per year, with around 25 million twenty-foot equivalent units (TEUs) containers throughput, and less than 200 cruise ships calls (Port of Rotterdam 2019, Port of Antwerp 2019, Port of Amsterdam 2019). Moreover, the importance of the research on this region is reinforced by the bunkering operations taking place covering more than 8% of the global demand with Rotterdam being the second biggest bunkering port in the world in terms of marine fuel sales(IHS Markit 2018). Furthermore, the recent IMO2020 sulfur cap has changed the bunkering market with the International Energy Agency (IEA) publishing forecasts with demand for heavy sulfur fuel oil (HSFO) dropping by 60%, demand for marine gas oil (MGO) doubling, and demand for very low sulfur fuel oil (VLSFO) covering approximately 25% of the “new” market (IEA 2019). Bunkering-port selection within this context is becoming challenging and is expected to become even harder with the recent developments of the southern European ports and railway infrastructure in Italy and Greece.

The methodology used to identify the effect of IMO2020 sulfur cap on the decision-making process of a bunkering port selection in the ARA region is a combination of primary and secondary research, along with a hybrid Fuzzy-Delphi-TOPSIS analysis method. The primary research involved interviews and questionnaires targeted on ship owners/operators (henceforth “operators”) and bunker suppliers involved in port activities at the ports of the ARA region. The secondary research involved a thorough coverage of the literature to date on the decision-making process of a bunkering port selection. The hybrid Fuzzy-Delphi-TOPSIS analysis method is a combination of Fuzzy-Delphi and Fuzzy TOPSIS methods used to quantify the qualitative information received from the interviewees. With this approach, the uncertainty of the decision space was limited, the individual expert opinions were kept intact, the performance of the ports could be quantified, and finally the rationality behind the MCDM process of a bunkering port selection process could be presented.

The analysis covers both the supply and demand side. The questionnaires were sent directly to contacts which were identified as experts in the bunkering business. Out of the initial 43 invitation for participation in this research, 17 questionnaires were received back. Out of these, eight questionnaires were completed by operators active in international trade, one questionnaire by an operator active in national trade, and eight questionnaires were completed by bunker suppliers. In order to perform the Fuzzy-TOPSIS, three experts were asked to contribute to this paper by submitting a second questionnaire. Since the decision-making process is performed by operators, it was decided that two of the experts would be from the demand side and one from the supply side. This approach was identified as the more accurate to analyze this topic.

This work provides a better understanding of the bunkering process both from the angle of the operators as well as the angle of the bunker suppliers. The audience will be provided with an evaluation of the importance of each KPF, they will be able to choose a bunkering port tailored to their needs and increase the efficiency of the bunkering process. From the angle of bunker suppliers, they will be able to evaluate their strategic advantages, they will identify their strengths and weaknesses, and new entrants will be able to evaluate the most efficient bunkering spot to enter the market. From a pure research perspective, this paper provides a first approach to understanding how the introduction of sulfur cap will affect the bunkering port selection process as factors like the availability of low sulfur bunker and the quantity of bunker are having an increased importance. Ergo, should any policy instrument impact the quality of fuel, as it is currently discussed within the framework of the International Maritime Organization (IMO), this methodology can be effortlessly replicated and adjusted to the needs of the specific case.

Some of the key findings suggest that operators consider the availability of low sulfur bunkers and the bunker quality as the most important factor when choosing a bunkering port. This partially reveals the effect of sulfur cap as availability and quality are now more important than price. Bunker suppliers considered bunker prices to be as important as bunker quality while they were not concerned about the availability of low sulfur bunkers. Regarding the performance of the ports, the port of Rotterdam ranked first as the most competitive port, followed by the port of Antwerp, and at the last place was the port of Amsterdam.

The paper is structured as follows: Sect. 2 presents a review of relevant papers covering the bunkering port decision making process, port competition, and the factors affecting port selection; Sect. 3 provides a detailed walkthrough of the methodology used; Sect. 4 analyzes the case of the ARA region and assesses the impact of sulfur cap on the competition of Northern European bunkering spots; and in the end, a conclusion is provided in Sect. 5 along with a brief summary of this paper.

## Literature review

Competition between ports and factors affecting competitiveness is a topic which has been widely discussed between researchers in the literature (Notteboom 2009; Tongzon 2007; Lirn et al. 2003; Yeo and Song 2005; Tai and Hwang 2005; Notteboom and Yap 2012) as it affects directly the maritime logistics’ value chain as well as investment decisions and financial planning of ships. Since the early days of exploring the impact of sulfur reduction on marine fuels’ market, focus has been placed on creating competitive cost structures while recently the importance of quality of services has emerged (Acosta et al. 2011). Moreover, Schinas (2016) provided a detailed analysis of the Sulfur Directive 1999/32/EC as amended, as well as a decision support system for the selection of sulfur compliant technology (Schinas and Stefanakos 2014). In an effort to minimize costs, solutions were proposed with the combination of speeds inside and outside Sulphur Emission Control Areas (SECA) (Doudnikoff and Lacoste 2014) or calculated a spread price (EUR231/tonne) based on which marine gas oil will have a higher net present value than scrubbers (Jiang et al. 2014). In other cases, researchers suggested a homogenous enforcement regime to prevent market distortion caused by the sulphur cap (Zis and Cullinane 2020). Finally, alternative approaches were also indicated with export credit facilities financing the higher price of compliance or acquisition of new green ships (Schinas et al. 2018), or the use of LNG as it is fully desulfurized (Schinas and Butler 2016).

Despite the extensive research on port competition and competitiveness, the research around the topic of bunkering competition and competitiveness is still limited. Port competition remains not a well-defined concept because of its complexity as the type of the port and the type of trade involved can affect the nature of competition (Notteboom and Yap 2012). Moreover, intra-port competition complicates further the analysis; Heaver (1995) suggests focusing on terminals and not on ports, when examining a competition strategy. Despite the broad number of publications on port competition and competitiveness that clearly focus on cargoes and logistics, hinterland connections and geography, there are few researchers who identified bunkering as an attribute of competition and competitiveness at ports. Another challenge appears regarding the importance of each factor affecting bunkering competition and competitiveness as importance is subjective depending mainly on the region (Acosta et al. 2011) and on the type of transport the vessel is performing (Sevgili and Zorba 2017). However, the most frequently cited factors affecting bunkering port choice are bunker price, bunker quality, geography, and level of services.

The factor affecting bunkering port choice cited by most scholars in this review is the bunker prices. Specifically, Lam et al. (2011) identified bunker price as the third most important factors affecting bunkering port selection between Singapore and Shanghai; Acosta et al. (2011) reported bunkering price as the dominant factor in their bunkering competitiveness survey, having the highest mean in their Likert-scale scores table; Yao et al. (2012) noted that bunkering prices is the main factor affecting bunkering port decision and suggested that the current practice of ships bunkering at few fixed ports can be improved; Vilhelmsen et al. (2013) added that not only bunker price is important, but also the window in which the bunker price remains valid is crucial especially for tramp shipping; Wang et al. (2014) also ranked bunker price as the most important factor in choosing the most optimal port between China, South Korea, and Japan; and Cariou and Notteboom (2011) noted that if the bunker price moves beyond the agreed band, then a recalculation of the bunker adjustment factor (BAF).

Bunker quality is also cited very often throughout the literature as a factor affecting bunkering port choice. Lam et al. (2011) found bunker quality to be the most important factor when selectin a bunkering port. This comes as a result of the high cost of debunkering operations. Debunkering is defined as “the removal of fuel from a vessel that was meant to be used for sailing. In some cases, this bunker fuel must be classified as waste, while in others it can be regarded as a product” (Inspectie Leefomgeving en Transport (ILT), 2020). Such operations can result in delays in ships’ schedule causing cascading effects on other ports and the commodities on board, leading to higher supply chain costs (Vernimmen et al. 2007). Similarly, in Wang et al. (2014), bunker quality is recognized as the second most important factor when selecting a bunkering port marginally below bunker price. According to the scholars, this can be explained as the quality of marine fuels can affect ship handling, engine operation, bunker consumption, and environmental impact of emissions (Wang et al. 2014). Finally, Acosta et al. (2011) also included fuel quality in their bunkering competitiveness survey. In their paper, even if fuel quality was supposedly the same in the three ports they examined as fuel were derived from the same CEPSA refinery at the Bay of Algeciras, still it was placed in position number eight as per its importance.

In addition to factors related directly to the product, bunkering decisions are also affected to a high extent to factors related to, the location of the port, and to geographical advantage the location can provide. The importance of geographical advantage of refueling area is based on fact that bunkering decisions are made based on scheduling and routing of vessels (Sevgili and Zorba 2017). Bunkering ports which are far away from loading/discharging ports can increase operational costs and cause instabilities in the supply chain. Tongzon (2002) identified geographical location as a rather important factor in choosing a port and described it as even more important than port charges. Similarly, Acosta et al. (2011) placed geographical advantage as the second most important factor in choosing a bunkering port even though, according to the authors, the result was biased by the positive perception of the region they were examining, i.e., the Strait of Gibraltar. Location was also an important factor in Ng’s (2006) paper in which, excluding bunkers’ price and quality, geographic location reported the third highest average significance score after monetary costs and time efficiency. The same researcher also noted that geographic location is a factor that can be divided into different scenarios. This is because, according to Ng (2006), a hub port that possesses advantages to a specific region is very likely to possess disadvantages on another region. Hence, the importance of geographic location as a factor of bunkering port choice is affected by the subjectivity of the region. Furthermore, Wang et al. (2014) have also pointed out the importance on geographic advantage in their study gathering the second highest score as a key performance factor after bunker price. Additionally, Yao et al. (2012) mentioned liner shipping companies are likely to choose the first port after a long journey as their bunkering port, increasing thus the importance of geographical advantage as a factor. Finally, Vilhelmsen et al. (2013) indicated that there are a few ports that dominate the bunker sales simply thanks to their strategic geographic location. Examples of such bunkering ports are Malta and Singapore (Oh and Karimi 2010).

In the recent years, there is also a trend of increased importance for the quality of services provided at the bunkering ports. Chang and Chen (2006) established a knowledge-based simulation model in order to evaluate the bunkering services, and specifically barges allocation, at the port of Kaohsiung. Acosta et al. (2011) spotted an increased in importance for quality services when choosing a bunkering port attributing it to the integration of ports within a larger logistics chain. This trend started appearing many years ago with Murphy and Hall (1995) reporting that shipping companies are willing to accept higher costs of service in exchange of higher quality services. In this context, they identified reliability, speedy delivery of goods, and considerations or preferences of transport operators as dominant in the selection of a port. To this extent, Pinder (1997) added that bunker services can extend the time spent on the port creating problems related to cargo handling. So bunkering services quality is an important factor for the port image and the customer satisfaction. Wong et al. (2008) verified the importance of reliability in their factors affecting the selection of ports and added that level of sophistication of assessment methods used to select transport operators has changed, the determinants of port selection has been reclassified, and the importance of cost has decreased. However, we need to take in mind that the latter publication took place in years were freight rates were at historical high. Guy and Urli (2006) also identified service quality as a major factor in choosing the optimal port along with port infrastructure, port location and costs. Similarly, Ng (2006) included efficiency and quality of service as major factors affecting port choice besides monetary factors.

Throughout the literature, scholars have identified more factors affecting the decision-making process of a bunkering port selection, based on the region they were investigating, and the time their papers were written. Some of the most cited are included in Table 1.

**Table 1:** Summary of bunkering port selection criteria as cited in scholars

*Articles*	*Criteria*
Acosta et al. (2011)	• Anchoring and docking availability
	• Simplicity/accessibility to port
	• Port tariffs,
	• Supply waiting time
	• Port Congestion
	• Port access waiting time
	• Ship inspection thoroughness
	• Prices of complementary services for fuel supply at berth and at anchorage
	• Simplicity of crew changes
	• Restrictive environmental regulations
	• Customs strictness
	• Clear and precise information about services
	• Hinterland proximity
	• Port security
	• Organizational tradition and capacity
	• Industrial disputes
Lam et al. (2011)	• Availability of low sulfur bunkers
	• Transparency
	• Reliability and punctuality of bunker suppliers
	• Bunkering facilities (adequacy and efficacy)
	• Government policies and incentives
	• Stability of political environment
Wang et al. (2014)	• Port bunker fuel capacity
	• Supply waiting time
	• Volume of containers
	• Safety of bunkering
	• Port bunker suppliers
	• Port bunkering supply regulations
	• Port tariffs
	• Information sharing among stakeholders
	• Port weather conditions
	• Efficiency of bunker supply
	• Environmental restrictions effects
	• Port time
Vilhelmsen et al. (2013)	• Port tariffs
	• Location of port

## Methodology

The proposed methodology aims at analyzing the process considered by operators in selecting bunkering ports by developing a multi-criteria decision making (MCDM) model. An MCDM process can used to explain decision-making problems with a diverse background having one aspect in common. That is multiple objectives and multiple criteria which usually come in conflict (Nădăban et al. 2016). The decision makers have to select and rank the factors affecting their choices based on the weight of the criteria. For the purpose of this paper, a hybrid form of MCDM process is going to applied on the bunkering market of the ARA region.

Selection of a bunkering port is a decision-making problem based on the preference of not only operators, but also of bunker suppliers who are entering a new market region. Another factor affecting the decision-making process of the shipping lines is the nature of the trade each company follows, i.e., liner or tramp. In this case, the decision-making process for tramp shipping is easier than for liner shipping. In the liner shipping case the decision makers cannot quantify easily the comparative ratios, making the preferential decision-making model uncertain (Tsai et al. 2010). Given the above, in this work widely-used applications of fuzzy theory is deployed, and more specifically Fuzzy Delphi method (FDM) proposed by Ishikawa et al. (1993), combined the extensions of Chen’s (2000) and Wang’s et al. (2018) to Technique for Order Performance by Similarity to Ideal Solution (TOPSIS) are applied in the next sections. The detailed steps of each phase are discussed as follows.

### Literature review and questionnaire preparation

The first step toward analyzing the decision-making process for the selection of bunkering ports was to perform a literature review, identify all possible key performance factors (KPFs), and include them in a questionnaire. Once all KPFs were identified, the questionnaire was sent to 43 contacts consisting mainly of shipping operators, 17 of which were received back. Out of these, eight were operators active in international trade, one was an operator active in national trade, and eight questionnaires were completed by bunker suppliers. The participants in the research were asked to rank the importance of each KPF by assigning a range from 1 (low importance) to 7 (high importance). In this paper, it was assumed that each group would prioritize differently the KPFs from less important to most important. Hence, a reliability analysis of respondents had to be performed in order to check how consistent the test was across raters. For this reason, Cronbach’s alpha was applied as the most common type of internal consistency reliability (Chong et al. 2012). The general acceptable values for Cronbach’s alpha is 0.6 to 0.7, with those are greater than 0.8 being viewed as highly reliable (Ursachi et al. 2015). Finally, the geometric mean (Eq. 1) was calculated in order to check the importance of each KPF and avoid the impact of extreme values:

1 $$\mathrm r=\sqrt{a_1\ast a_2\dots\ast a_i,}\mathrm i=1,2,\dots,17$$

where a represents each KPF.

### Fuzzy Delphi method

The traditional Delphi method has faced many problems in its application. Specifically, the objectivity of the interviewees is questionable as they can be influenced by the survey organizers’ opinion (Kuo and Chen 2008), the generalization of the results to a wider population because of the size of the sample (Schmidt et al. 2001), and its inability to transform experts’ judgements into quantitative data (Wang and Lin 2008). For these reasons, this paper chose to adopt Ishikawa et al. (1993) Fuzzy Delphi method with the modifications of Kuo and Chen (2008) to denote expert consensus with geometric means. The process is demonstrated as follows:

- Convert expert opinions collected from questionnaires into quantitative data by selecting the KPF that were above the threshold r and applying to them a 10-point scale level of importance (Table 2) based on the answers submitted.
- Create the triangular fuzzy number (TFN) *W*_ij_ as shown in Eq. 2 where *i* indicates the alternatives (in our case the ports); *j* indicates the criteria (in our case the factors); *a*_ij_ indicates the lowest appraisal value submitted by the interviewees; *b*_ij_ indicates the geometric mean of all interviewees; *c*_ij_ indicates the top of all interviewees’ appraisal value; and *M*_ijk_ indicates the appraisal value of the kth expert.
  2 $${W}_{ij}=({a}_{ij},{b}_{ij}{,c}_{ij})$$

where:

$${a}_{ij}=\mathrm{min}\left({M}_{ijk}\right)$$

$$b_{ij}=\sqrt[n]{\Pi_{i=1}^n}M_{ijk},$$

$${c}_{ij}=\mathrm{max}\left({M}_{ijk}\right)$$

and:

**Table 2:** Linguistic variables for the preference of each alternative (Buyukozkan and Cirfi 2012)

^Linguistic scale^	Fuzzy score
^Good (G)^	(8, 9, 10)
^Medium fair (MF)^	(6, 7, 8)
^Fair (F)^	(4, 5, 6)
^Medium poor (MP)^	(2, 3, 4)
^Poor (P)^	(1, 1, 2)

$${a}_{ij}\le {b}_{ij}\le {c}_{ij}$$

### Fuzzy TOPSIS

TOPSIS was introduced by Hwang and Yoon (1981) and became the most identified technique for solving multi-criteria decision-making problems. Since then, there were numerous scholars who proposed extensions to TOPSIS. This paper is going to use a combination of Chen’s (2000) and Wang’s et al. (2018) extensions to bring the methodology closer to the bunkering market.

Hence, once TFNs have been calculated, the importance of each port needs to be determined by choosing the fuzzy linguistic values using the fuzzy linguistic rating (in our case 0–10 as seen in Table 2).

In case a KPF has a crisp quantity value (0,1), a transformation to fuzzy number will be needed. In this case, if the crisp quantity of port *A*_*n*_ is ${R}_{ij}^{0}=({a}_{ij}^{0},{b}_{ij}^{0},{c}_{ij}^{0})$ where ${a}_{\mathrm{ij}}^{\mathrm{o}}= {b}_{\mathrm{ij}}^{\mathrm{o}}={c}_{\mathrm{ij}}^{\mathrm{o}}$ then the KPFs must be transformed into dimensionless numbers in order to be compatible with the linguistic numbers of the KPFs. In this paper, Choo (2009) transform method will be used as cited in Wang et al. (2018) as explained in Eqs. 3 and 4.

3 $${\overline{R} }_{ij}= \left\{{R}_{ij}^{o}/\underset{i}{\mathrm{max}}\left\{{c}_{ij}^{o}\right\}\right\} \times 10$$

4 $${\overline{R} }_{ij}= \left\{\underset{i}{\mathrm{min}}\left\{{a}_{ij}^{o}\right\}{/R}_{ij}^{o}\right\} \times 10$$

where:

i) $\underset{i}{\mathrm{max}}\left\{{c}_{ij}^{o}\right\}>0$, ${\overline{R} }_{ij}$ denotes the transformed fuzzy number of objective benefit ${R}_{ij}^{o}$, ${\overline{R} }_{ij}$ becomes larger when the objective benefit ${R}_{ij}^{o}$ is larger.
ii) ($\underset{i}{\mathrm{min}}\left\{{a}_{ij}^{o}\right\}>0$, ${\overline{R} }_{ij}$ denotes the transformed fuzzy number of objective benefit ${R}_{ij}^{o}$, ${\overline{R} }_{ij}$ becomes smaller when the objective cost ${R}_{ij}^{o}$ is larger.

Moving on, this paper calculated the normalized global rating for each KPF by multiplying each KPF’s fuzzy weigh with the fuzzy rating that the same KPF had for each port.

5 $${\mathrm{v}}_{ij}={x}_{ij}*{\mathrm{w}}_{j}$$

where *X*_ij_ is the TFN playing the role of fuzzy weight; *w*_j_ is obtained from the fuzzy Delphi Method via expert questionnaires.

Furthermore, the fuzzy positive ideal solution (FPIS, A^+^) and the fuzzy negative ideal solution (FNIS, A^−^) needed to be determined (Eqs. 6 and 7) and then calculate the distance of each port from A^+^ and A^−^ (Eqs. 8 and 9), by subtracting the position of each port from D + and D − (Eq. 10).

6 $${\mathrm{A}}^{+}=\left\{{\mathrm{v}}_{1}^{+},\dots ,{\mathrm{v}}_{\mathrm{i}}^{+}\right\}$$

7 $${\mathrm{A}}^{-}=\left\{{\mathrm{v}}_{1}^{-},\dots ,{\mathrm{v}}_{\mathrm{i}}^{-}\right\}$$

8 $${\mathrm{D}}_{\mathrm{i}}^{+}=\sum\nolimits_{\mathrm{j}=1}^{\mathrm{m}}\mathrm{d}\left({\stackrel{\sim }{\mathrm{V}}}_{\mathrm{ij}},{\stackrel{\sim }{\mathrm{V}}}_{\mathrm{i}}\right),\mathrm{i}=\mathrm{1,2},\dots ,\mathrm{n}$$

9 $${\mathrm{D}}_{\mathrm{i}}^{-}=\sum\nolimits_{\mathrm{j}=1}^{\mathrm{m}}\mathrm{d}\left({\stackrel{\sim }{\mathrm{V}}}_{\mathrm{ij}},{\stackrel{\sim }{\mathrm{V}}}_{\mathrm{i}}\right),\mathrm{j}=\mathrm{1,2},\dots ,\mathrm{m}$$

10 $$d\left(\stackrel{\sim }{\mathrm{a}},\stackrel{\sim }{\mathrm{b}}\right)=\sqrt{(1/3)\left[{\left({\mathrm{a}}_{1}-{\mathrm{b}}_{1}\right)}^{2}+{\left({\mathrm{a}}_{2}-{\mathrm{b}}_{2}\right)}^{2}+{\left({\mathrm{a}}_{3}-{\mathrm{b}}_{3}\right)}^{2}\right]}$$

where $\tilde{a }$ and $\tilde{b }$ are two TFNs, shown by the triplets (${a}_{1},{a}_{2},{a}_{3}$) and (${b}_{1},{b}_{2},{b}_{3}$).

Finally, the relative closeness of each port to the ideal solution needed to be calculated. The relative closeness of the alternative ${A}_{i}$ in relation to ${A}^{+}$ is characterized as below:

11 $${FC}_{i}={D}_{i}^{-}/{(D}_{i}^{*}+{D}_{i}^{-}), i=\mathrm{1,2},\dots ,n$$

### Validation through statistical evidence

Considering bunker price data available in the industry, a brief statistical analysis is performed, that will validate the findings and results of the previous steps. The data source used in the present work are the daily time series of the HSFO (380cSt) and MGO in Rotterdam, as published by Shipping Intelligence Network of Clarksons. Rotterdam is the main bunkering spot in the region, so the prices in ARA region as well as of related ports, such as of Hamburg, as generally pegged with an almost fixed surcharge to the ones of Rotterdam. The lack of sufficient data for low-sulfur (LS) HFO and marine diesel oil (MDO) did not permit the performance of similar statistical analysis. Nevertheless, it is noted that HSHFO results relate to ships equipped with exhaust gas cleaning system (EGCS), i.e., a scrubber or similar technology, while the MDO and MGO prices to ships without an EGCS installed.

**Fig. 1:**
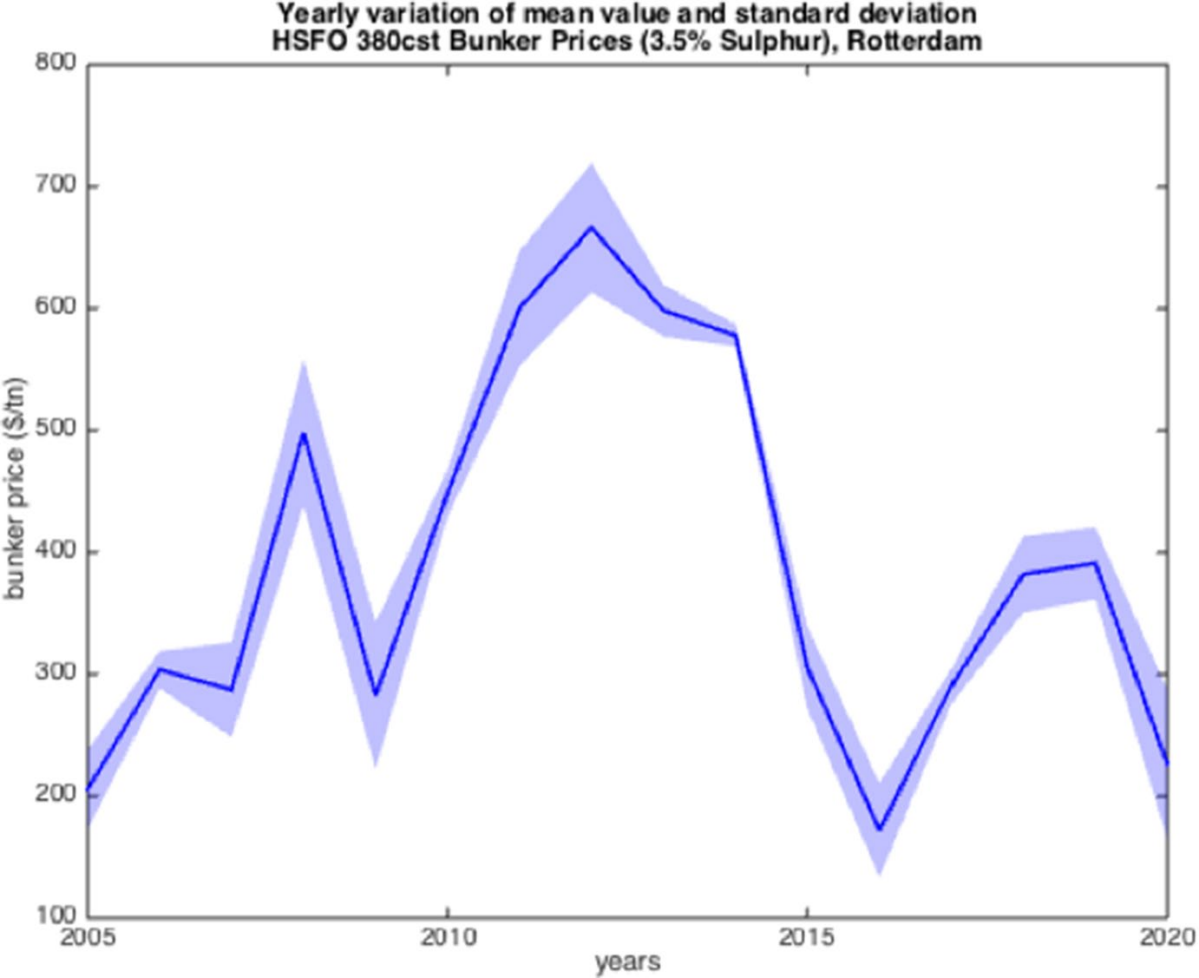
Yearly variation of mean and standard deviation HSFO 2005–2020

**Fig. 2:**
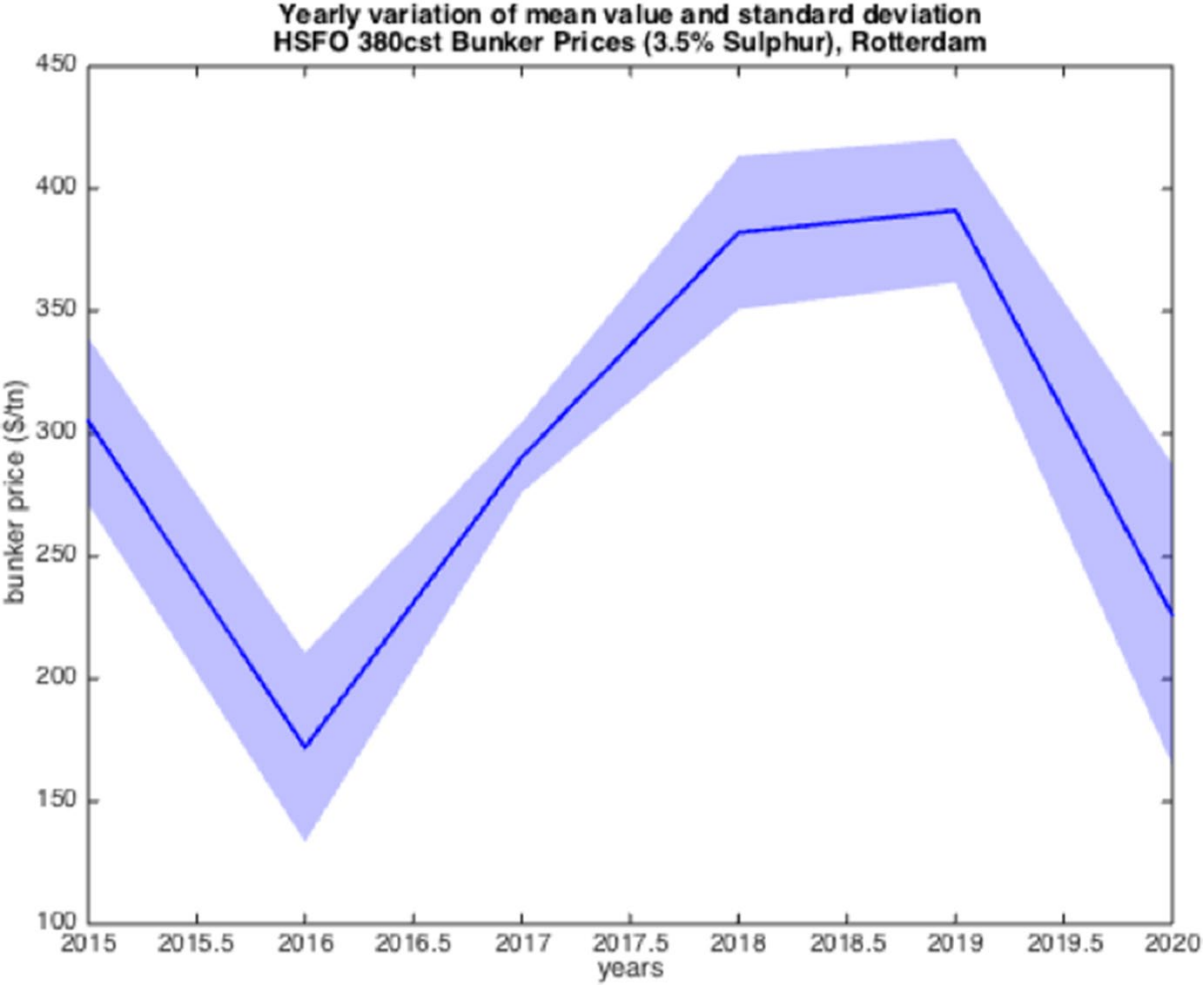
Yearly variation of mean and standard deviation HSFO 2015–2020

**Fig. 3:**
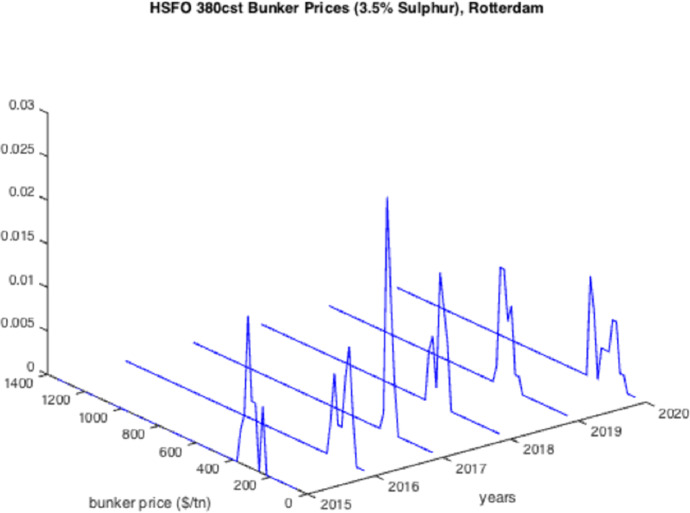
Histograms of the HSFO prices 2015–2020

**Fig. 4:**
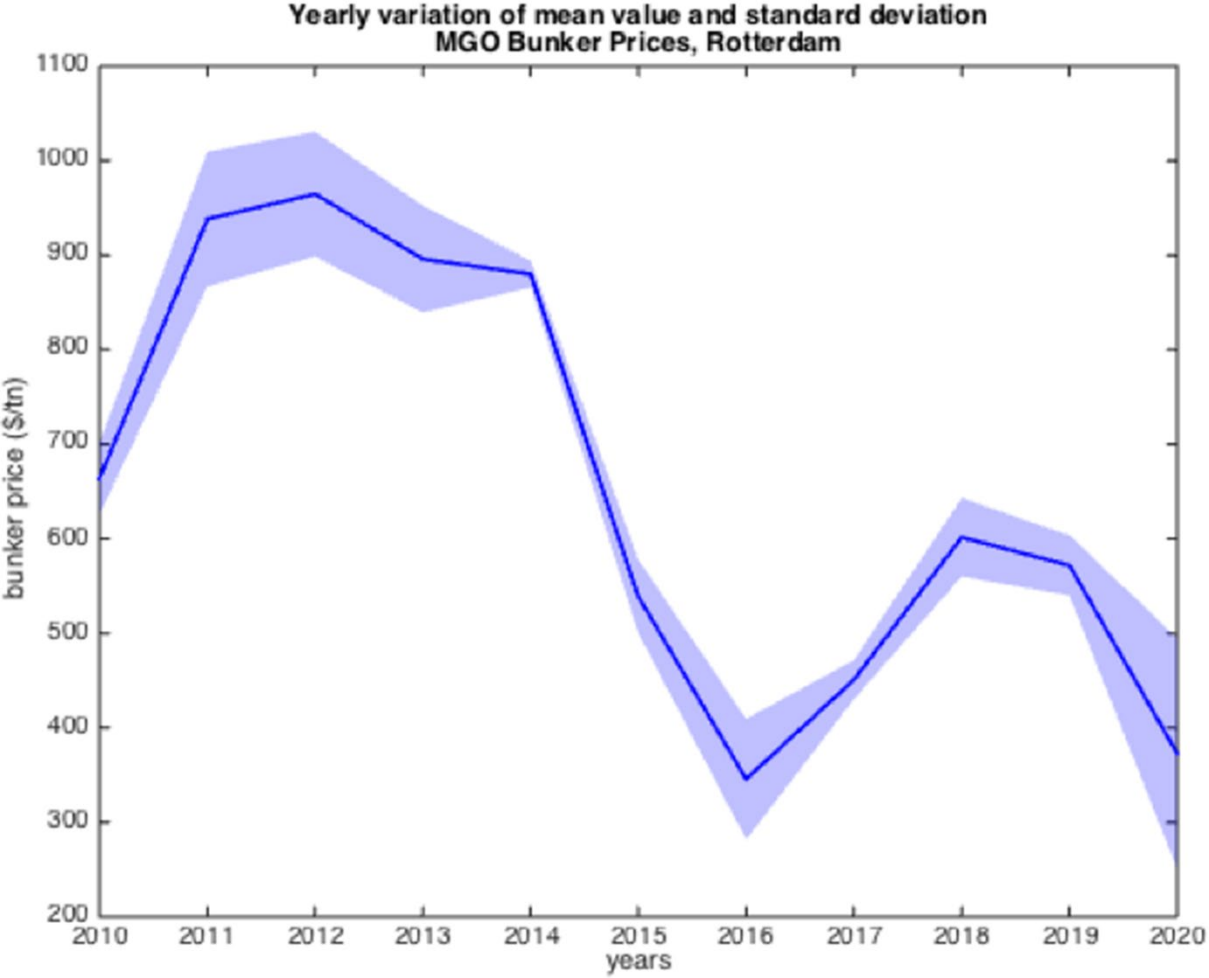
Yearly variation of mean and standard deviation MGO 2010–2020

**Fig. 5:**
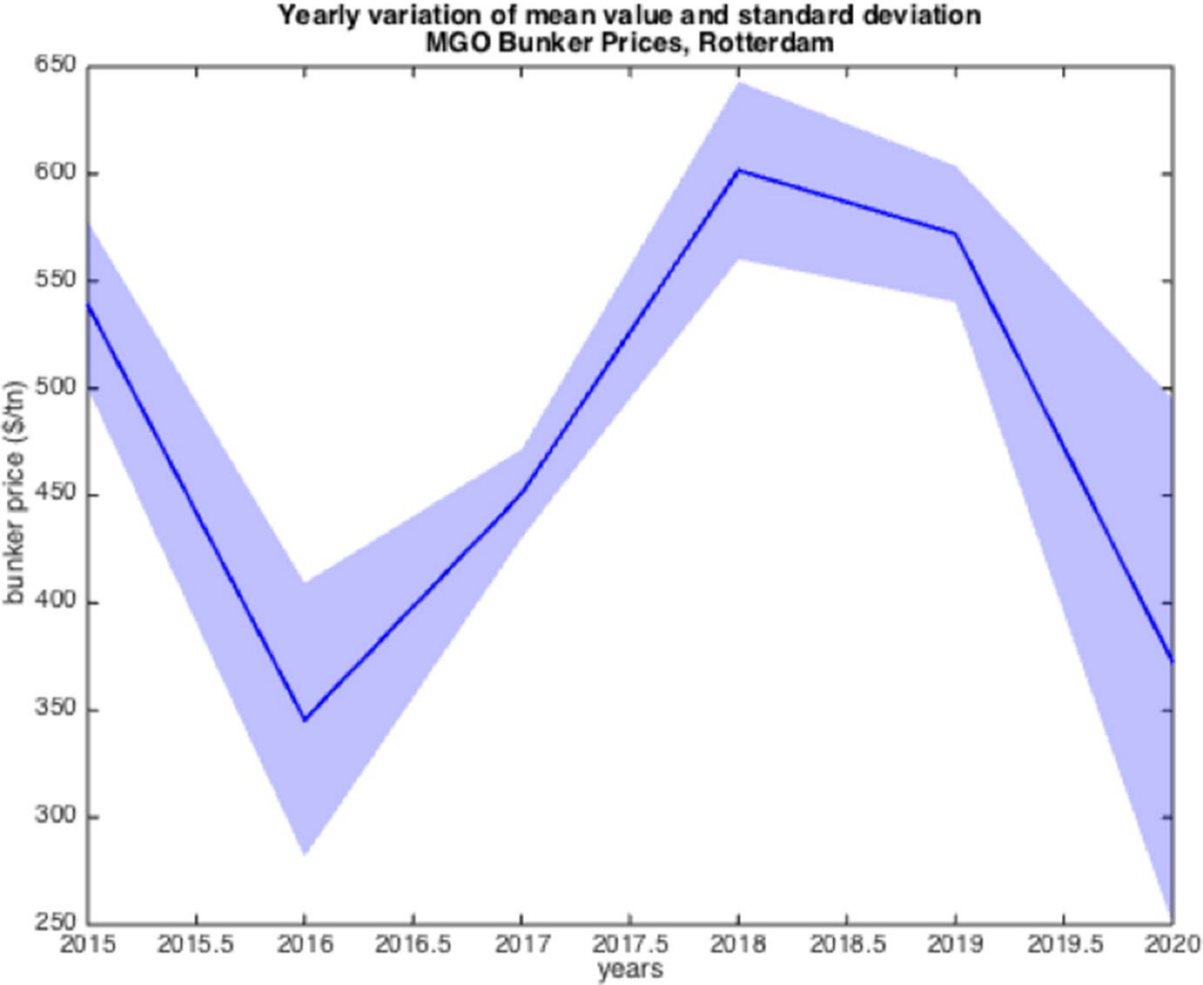
Yearly variation of mean and standard deviation MGO 2015–2020

**Fig. 6:**
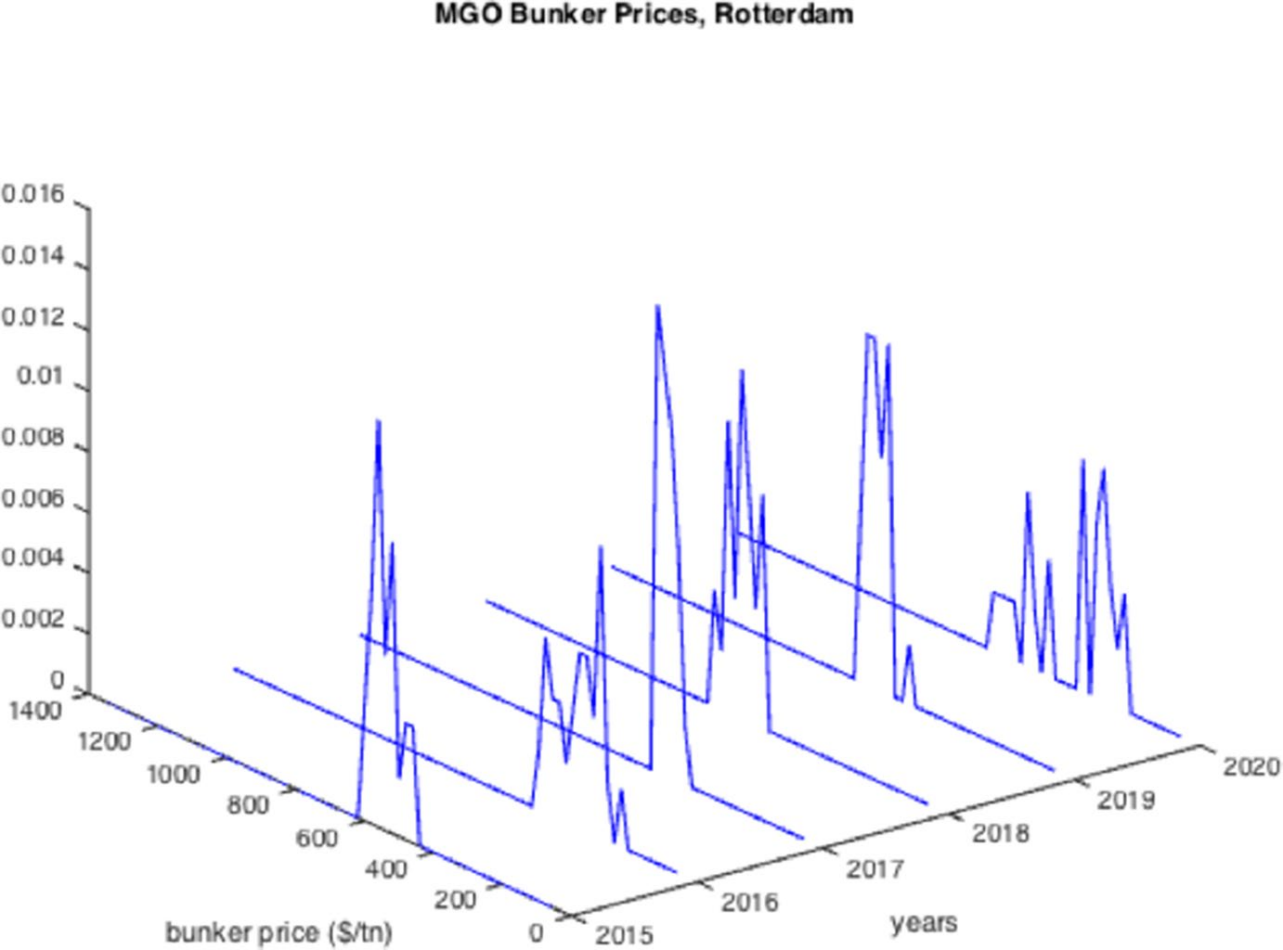
Histograms of the MGO prices 2015–2020

The resulted graphs from the statistical analysis below focus on the first semester of each year from 2005 to 2020; in Appendix Table 10, a detailed statistical Analysis of the HSFO data in Rotterdam for every year since 1990 is provided for comparison purposes as well as for further elaboration. The analysis of the time-series for HSFO and MGO suggests (see Fig. 1, 2, 3, 4, 5, 6, and Appendix Table 10) that 2020 data do not deviate from the statistical pattern of the last years. There is no sign of higher prices than statistically expected. This might be the outcome of the global economic recession in the first semester of 2020 due to the pandemic, but such an issue would have emerged in the qualitative analysis of the questionnaires and of the interviews.

Finally, the annual 2020 Fuel Oil Quality and Safety Survey of BIMCO (2020) does not identify any price issues in the transition to IMO 2020 levels, following similar methodology.

## The case of the ARA region

In this section of the paper, a case study of bunkering spots in Northern Europe is examined. Specifically, the ports of the ARA region were taken under consideration in order to compare their competitiveness determinants. This is because the region is identified as the most important hub for maritime activity in Northern Europe with Rotterdam, Antwerp, and Hamburg accounting for more than 40% of the total TEUs in European ports in 2018 (Eurostat 2020). Furthermore, the hub is the second biggest bunkering hub (more than 8% of total supply) in the world after Singapore (IHS Markit, 2018), while 60% of Europe’s GDP is located within 500 km radius, making it an important hub for the European market as well. This, in conjunction with the increase in port calls and total throughput year on year reinforce the importance of the ARA region as a bunkering hub.

### Selection of KPFs—Delphi method

At a first stage, this paper identified 27 key performance factors connected to the decision-making process of a bunkering-port selection, after going over the literature and putting together the most significant factors that scholars had identified in the past. These 27 KPFs were included in an electronic questionnaire that was sent to 43 individuals with more than 10 years of experience in shipping operations covering both the supply market and the demand market. Seventeen answers were received, ten of which came from people working in top shipping companies, and seven working in bunkering companies. The respondents were not required to be focused in the ARA region as the goal of the questionnaire was to identify a global trend in what drives the decisions-process of bunkering-port selection.

Once all answers were received, Cronbach’s alpha was applied to test the reliability of the answers before moving on with the selection of KPF for this paper. The value of ~ 0.8175 was obtained making the answers highly reliable. To determine the importance of each individual factor, a 7-point scale was introduced, based on which every respondent had to put a score to each KPF with one being the lowest score and 7 being the highest score. Once all answers were collected the geometric mean of each KPF was calculated (Table 3). For example, for the KPF “Availability of low sulfur bunkers” the geometric mean was:

**Table 3:** Key performance factors

Factors	Geometric mean value
Availability of low sulfur bunkers	6.68
Bunker quality	6.65
Bunker price	6.52
Reliability, punctuality, and safety of bunkering services	6.49
Usage and availability of barges	6.10
Bunker quantity	6.08
Clear and precise information about services	5.98
Transparency (corruption-free)	5.87
Supply waiting time	5.58
Location of port	5.57
Geographical advantage	5.56
Duration of supply	5.54
Bunkering rules of port	5.46
Bunkering facilities (adequacy and efficacy)	5.24
Prices of complementary services for fuel supply at anchorage	5.20
Anchoring and docking availability	5.18
Port congestion	5.15
Quality of bunkering services (e.g.pumping rates)	5.11
Navigational availability (night navigation)	5.09
Port tariffs	4.88
Presence of restrictive environmental regulations	4.79
Simplicity/accessibility to port	4.75
Ship inspection thoroughness	4.71
Government policies (e.g.quality control) and incentives	4.58
Prices of complementary services for fuel supply at berth (pilotage mooring etc.)	4.53
Customs strictness	4.30
Simplicity of crew changes	4.17

$$=\sqrt{7*5*7*6*7*7*7*7*6*7*6*7*7*7*7*7*7}=6.6786$$

The initial goal was to exclude all KPFs with score lowest than the threshold *r* = 4. However, all KPFs scored above 4 making all KPFs performance evaluation criteria capable of affecting the bunkering port selection process.

**Fig. 7:**
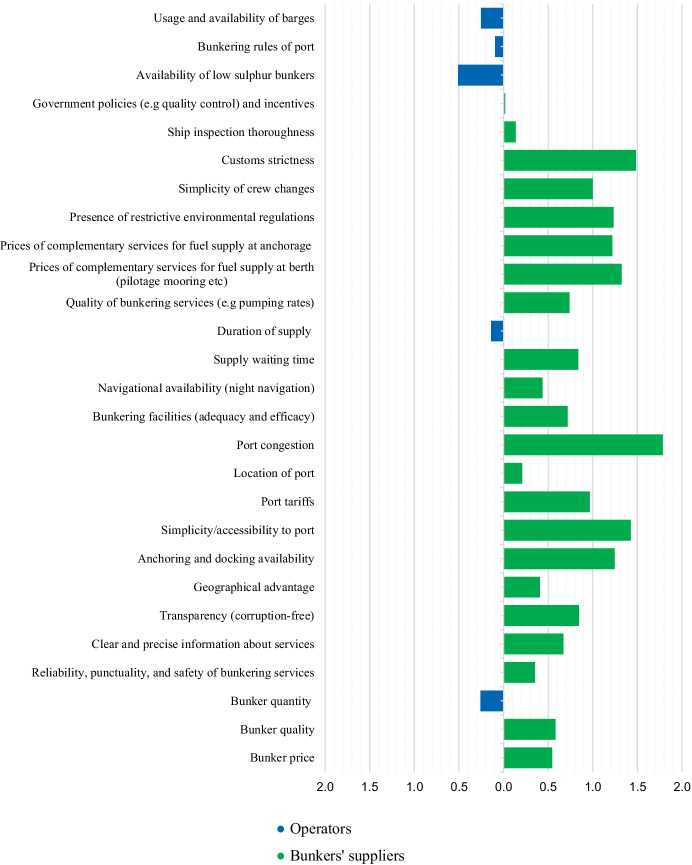
KPF geometric mean—operators vs. bunker suppliers

Before moving forward, there is the need to highlight the difference between the answers that were received from operators and bunker suppliers. Comparing the two groups, KPF “availability of low sulfur bunkers” reported the highest result in the operators’ group while “bunker quantity” was also high in the list. This indicates that operators are highly concerned about whether they can find enough bunkers to comply with IMO2020, but they are also concerned whether the suppliers are able to accommodate specific quantity parcels or all needs of the shipping company. The received responses suggest that operators value the KPFs connected with the supply itself higher than the KPFs which are directly connected to the bunkers (i.e., quality and price). This can lead to the conclusion that if needed, operators are willing to pay higher prices for bunkers as long as they can be reassured that the next bunkering port has either enough availability of low sulfur bunkers or the specific amount the operators need. On the other hand, bunker suppliers valued bunker quality as the most important KPF for which the response was a unanimous 7 (i.e., the highest possible score). Bunker prices followed closely in the second place while KPFs associated with the supply service itself followed up. Interestingly, the KPF “availability of low sulfur bunkers” was at the sixth place showing that there is an inconsistency in how the two groups perceive the new bunkering market after the effect of IMO2020. This can be better illustrated in Fig. 7 which depicts the difference between the geometric mean of each KPF for each group. The bars on the left of the y axis show that operators valued the respective KPF higher than bunker suppliers and vice versa.

### Determination of KPFs’ fuzzy weights

Since the preference of a bunkering port is the decision of the shipping companies and not the bunker suppliers, this paper isolated the answers of three professionals working in shipping companies and converted the Likert scale answers from a 7-point to a 10-point scale answers using SPSS (IBM Support, 2020) and specifically the following equation:

12 $$Y=\left(B-A\right)*\frac{x-a}{b-a}+A$$

where *Y* indicates the converted result, x is the result in the 7-point scale, *a* and *b* are the minimum and the maximum of the 7-point Liker scale respectively (i.e., *a* = 1 and *b* = 7), *A* and *B* and the minimum and the maximum of the new 10-point scale (i.e., *A* = 1 and *B* = 10).

Once the KPFs were identified and the shipping employees’ responses were converted into a 10-point scale system, a second questionnaire was handed out to the three experts who are actively working in the ARA region. The three experts were chosen according to how active they are in the ARA region. Since the perspectives of both the suppliers and buyers of bunkers was needed to be captured, one respondent was from the supply side and two respondents were working in important shipping companies. Under the Fuzzy Delphi method, the triangular fuzzy numbers of KPFs can be collected following Eqs. (2) to (5). Using the KPF bunker price as an example:

$${a}_{ij}=\mathrm{min}\left({M}_{ijk}\right)=\mathrm{min}\left(10.0, 10.0, 7.0\right)=7.0$$

$${b}_{ij}=\sqrt[n]{{\Pi }_{i=1}^{n}}{M}_{ijk}=\sqrt[3]{10.0*10.0*7.0}=8.8$$

$${c}_{ij}=\mathrm{max}\left({M}_{ijk}\right)=\mathrm{max}\left(10.0, 10.0, 7.0\right)=10.0$$

Once *a*_ij_, *b*_ij_, *c*_ij_, have been calculated, the TFN for the KPF bunker price will set as (7.0, 8.8, 10.0). In this survey, the three experts identified the KPFs availability of low sulfur bunkers, bunker quality, duration of supply, bunker price, and bunker quantity as the most important factors in choosing a bunkering port with an geometric mean of more than 8.5 out of 10 (availability of low sulfur being the only one with a score of 10 out of 10).

### Fuzzy TOPSIS analysis on the ARA ports

In this analysis, 27 KPF have been identified as important in the decision-making process of a bunkering port selection, some of which can be identified as quantifiable, meaning that data were available in order to assess the fuzzy rating of each port for these specific factors. As an example, for the KPF bunker price, instead of focusing on the qualitative and subjective answers submitted in the questionnaire, the relevant bunker prices were collected, and compared (Table 4). In this way, instead of depending on the subjective decisions of the experts, it observed the objective data provided by the port authorities. Since only one KPF was attributed to bunker prices, but there were data available for three types of bunkers, the average was taken for each port. For example, instead of having one KPF for IFO380, one KPF for VLSFO, and one KPF for MGO, the three types were aggregated and divided by three. Thus, the average bunker price for Amsterdam, Rotterdam, and Antwerp was set to $191.9/ton, $185.6/ton, and $180.2/ton respectively. This result was then converted into a fuzzy rating for each port. The second KPF that was replaces with objective data was “bunkering facilities (adequacy and efficacy)” as data were collected from port authorities regarding the number of bunker suppliers, lube suppliers, and barge operators. More information on objective KPFs can be found in Table 4.

**Table 4:** Objective KPFs’ fuzzy ratings

Objective KPFs	Quantity	Fuzzy rating
***IFO380 price***		
Amsterdam	148.0	(8.9, 8.9, 8.9)
Rotterdam	132.3	(10.0, 10.0, 10.0)
Antwerp	134.1	(9.9, 9.9, 9.9)
***VLSFO price***		
Amsterdam	195.2	(9.5, 9.5, 9.5)
Rotterdam	192.0	(9.7, 9.7, 9.7)
Antwerp	185.7	(10.0, 10.0, 10.0)
***MGO price***		
Amsterdam	232.6	(9.5, 9.5, 9.5)
Rotterdam	232.4	(9.5, 9.5, 9.5)
Antwerp	220.9	(10.0, 10.0, 10.0)
***Bunker suppliers***		
Amsterdam	24	(8.0, 8.0, 8.0)
Rotterdam	30	(10.0, 10.0, 10.0)
Antwerp	25	(8.3, 8.3, 8.3)

Once fuzzy weights and fuzzy ratings were collected, the normalized global rating was calculated by multiplying each KPF’s fuzzy weigh with its fuzzy rating (Eq. 5). Additionally, the positive-ideal solution (A +) and negative-ideal solution (A −) for each KPF was obtained using Eqs. 6 and 7. Having calculated the global ratings and the ideal solutions, this paper calculated the distance (D +) from the positive-ideal solution, and the distance (D −) from the negative-ideal solution for each port as well as the relative closeness (FC) of each port to the ideal solution (Eqs. 8, 9, 10, and 11). An example of this calculation can be seen in Table 5 and Table 6. In the former, the example of the KPF “bunker price” is presented while in the latter contains the aggregation of all KPFs distances for each port. A detailed list with the performance of each port can be found in Appendix Tables 8 and 9.

**Table 5:** Ideal distances of KPFs

Ports	D +	D-	FC	Ranking
*KPF 1: bunker price*				
Amsterdam	5.312	0	0.00	3
Rotterdam	2.505	2.808	0.53	2
Antwerp	0	5.312	1.00	1

**Table 6:** Final ranking of ports

Ports	D +	D −	FC	Ranking
Amsterdam	243.533	16.516	0.064	3
Rotterdam	2.505	253.404	0.990	1
Antwerp	150.296	119.474	0.443	2

It needs to be pointed out here that for 11 KPFs the distances from the positive ideal solution and the negative ideal solution was 0 as the three experts submitted equal scores for each port. This means that the interviewees believe the three ports performed equally well or equally bad for these 10 KPFs.^1^

According to the results of this research (Table 6), the most competitive port in the ARA region is the port of Rotterdam scoring a relative closeness to the ideal solution of 0.99 followed by the port of Antwerp with a relative closeness of 0.44 and last was the port of Amsterdam with a result of 0.06. Unfortunately, this result cannot be verified by objective data as only the port of Rotterdam reports volume of sales for bunkers. However, with the port of Rotterdam outperforming by 30 percentage points the port of Antwerp with respect to total yearly throughput, and number of ship calls, we can safely say that Rotterdam wins bunkering competition in the ARA region.

**Fig. 8:**
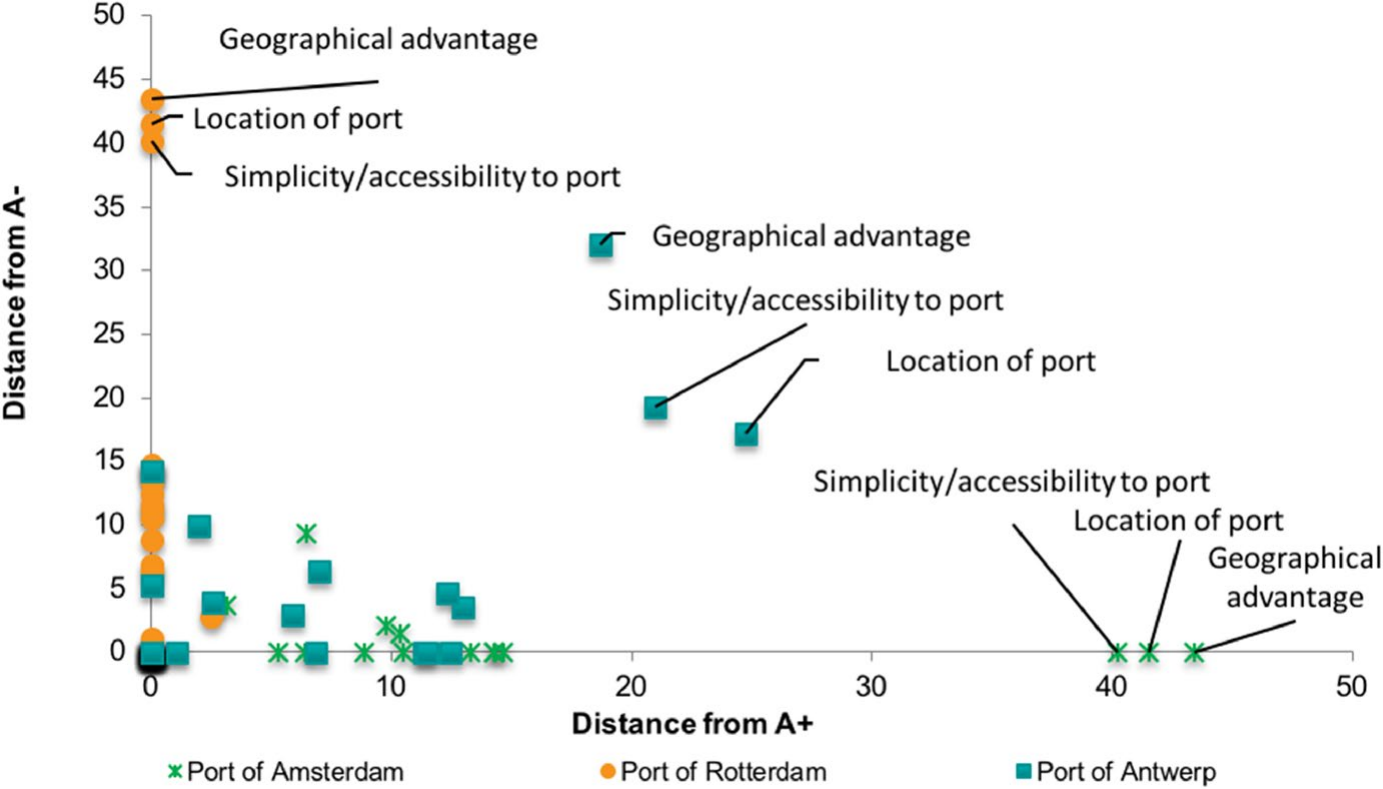
Distanceof KPFs from positive and negative ideal solutions

Analyzing each port individually, one of the main factors affecting the competitive environment in the region is related to location. Specifically, the port of Rotterdam had the highest distance from the negative ideal solution, the port of Amsterdam vice versa had the biggest distance from the positive ideal solution, and the port of Antwerp was somewhere in between. This is illustrated in Fig. 8 where a summary of the performance of the three ports can be seen in an XY Scatter chart. The closer each KPF is to the *Y*-axis the better the performance of the port and the highest within *Y*-axis, the biggest the competitive advantage. As an example, the port of Rotterdam had the best performance for all KPFs but bunker price. For this reason, all its entries are on the *Y*-axis apart from one. Moreover, the port of Rotterdam had the biggest competitive advantage in the KPFs geographic advantage, location of port, and simplicity/accessibility to port which is why these three KPFs where higher on the *Y*-axis. The opposite applies for the *X*-axis.

Looking deeper into each port, the port of Amsterdam is inferior to the rest of the ports in the ARA region for every single KPF. Apart from the aforementioned performance related to location, the port reported a large distance from the positive ideal solution related to supply waiting time, availability of low sulfur bunkers, simplicity of crew changes, anchoring and docking availability, and clear and precise information about services. On the contrary, it was close to the positive ideal solution on the quality of bunkering services, bunker quality, bunker prices, port congestion, and reliability, punctuality, and safety of bunkering services.

Regarding the port of Rotterdam, the only KPF that the port reported an inferior performance compared to the rest of the ports is bunker prices. For the period for which bunker prices were collected (i.e., 01/04/2020–01/05/2020), the port of Rotterdam reported the highest average price for IFO380, while for VLSFO and MGO its average price was about $10/ton higher than the port of Antwerp.

Finally, the port of Antwerp managed to compete with the top performer on bunker quantity, bunker quality, bunkering facilities (adequacy and efficacy), port congestion, anchoring and docking availability, and quality of bunkering services (e.g., pumping rates) as its distance for these KPFs was relatively small. However, the port did not manage to keep up with the competition on supply waiting time, reliability, punctuality, and safety of bunkering services, simplicity of crew changes, navigational availability (night navigation), and clear and precise information about services.

As mentioned earlier, there were also 11 KPFs that the three ports reported identical results. Out of these 11 KPFs that were presented earlier, the three ports reported a fair performance only for one KPF while for the rest the performance fluctuated between medium good and good (Table 7).

**Table 7:** KPFs with equal results for the three ports

Poor	Medium poor	Fair	Medium good	Good
		Prices of complementary services for fuel supply at anchorage	Transparency (corruption free)	Duration of supply
			Port tariffs	Presence of restrictive environmental regulations
			Prices of complementary services for fuel supply at berth (pilotage mooring etc.)	Simplicity of crew changes
			Customs strictness	Government policies (e.g.quality control) and incentives
				Ship inspection thoroughness
				Bunkering rules of port

Examining the three ports in a more general way, and taking always mind what can the ports of Antwerp and Amsterdam change in order to be able and win the competition against the port of Rotterdam, the following main conclusion can be drawn:

- Because the importance of the KPFs connected to location to the final results, the port of Rotterdam has the biggest competitive advantage against the port of Antwerp and the port of Amsterdam, a competitive advantage that is difficult to be outperformed. Hence, we expect the port of Rotterdam to remain the leader in the ARA region.
- The port of Amsterdam and the port of Antwerp need to plan on closing the gap with the port of Rotterdam by focusing on KPFs that are related to services provided. Specifically, the two ports need to focus on the KPFs in which their performance was identical fair and medium good compared to the port of Rotterdam (Table 7).
- With respect to the KPFs connected with environmental regulations up to a certain extent, these are regulated by the international law hence no significant changes ca be implemented. However, there are still some differences between the three ports since they are in different countries. That said, the Dutch government had started a discussion in 2015 regarding unwanted chemical substances, the debunkering operations (M.H. Schultz van Haegen, 2015). Such legislations can have an impact on the competition between the two countries as such processes can increase the cost of bunkering operations (transferring a percentage of the cost increase to bunker prices) but also, they can an impact on bunker quality, which can explain the reason, the port of Antwerp had the lowest bunker prices but also scored low on bunker quality.

## Conclusion

This paper assesses the effect of sulfur cap on Northern European bunkering spots. It also presents an exploratory analysis of bunkering competition and competitiveness at the ports of the ARA region. With bunkering costs representing approximately 50% on average of total operating costs of operators, choosing the optimal bunkering spot is an important factor in shipping operations as it can have a great influence in operators’ profits.

The outcomes of this work can be summarized as follows, given the respective interest of stakeholders:

1. Operators can better assess the KPFs and prioritize their choice of bunkering spot.
2. Bunker suppliers can better assess strengths and weaknesses of every bunkering spot (location attributes) and can formulate strategies to increase the attractiveness of their offer.
3. Researchers and policy-makers can replicate the developed methodology for assessing the impact of any new policy instrument related to fuel and its quality.

It is plausible to argue that the identified KPFs might serve also as parameters in other methodological approaches, whether qualitive or quantitative ones. As an example, bunker suppliers can consider these KPFs when developing strengths-weaknesses-opportunities and threats (SWOT) analyses for their commercial needs. Furthermore, the application of fuzzy-TOPSIS as well as the use of the developed questionnaires in relevant future research projects may support the effort and derive results for wider regions, e.g., of the East or the West Mediterranean. In this regard, policy-makers can assess the impact of instruments at a regional or any other level of interest. Hence, this work can transparently serve diverse needs and objectives of stakeholders with streamlined or conflicting interests.

In the first section, it was identified that bunkering-port selection is a multidimensional problem with many conflicting parameters. The factors that affect the choice can range from the type of operations (i.e., liner shipping has more limitations than tramp shipping) to more direct factors like the location of the ports, the price and quality of bunkers, and the quality of services provided. Since operators in tramp shipping can deviate with more freedom this paper focused mainly on liner shipping. Even though this MCDM process is performed mainly through advanced software, there is still a need for a holistic performance analysis of bunkering spots in order to create a benchmarking framework. This is because such software can work optimally when vessels’ schedule is accurate ignoring liner shipping companies’ preferences. Hence, this paper used Fuzzy-Delphi-TOPSIS methodology after adjusting it to the Northern European region and after including the latest market developments (sulfur cap, COVID-19, oil price wars) under consideration.

The results reveal that the KPFs availability of low sulfur bunkers, bunker quality, bunker price, reliability, punctuality, and safety of bunkering services, usage and availability of barges, and bunker quantity (i.e., ability to accommodate specific quantity parcels) are regarded as the most important in selecting a bunkering port. Operators consider the availability of low sulfur bunkers and the bunker quality as the most important factors when choosing a bunkering port. On the other side, the bunkers suppliers considered bunker prices to be as important as bunker quality while they were not concerned about the availability of low sulfur bunkers. The findings of the analysis are streamlined with the BIMCO report (2020) as well as with the results of statistical analysis of the price time-series of Rotterdam, hence confirming the sound methodological approach as well as the sufficiency of the gathered information.

Regarding the performance of the ports, the port of Rotterdam ranked first as the most competitive port, followed by the port of Antwerp, and at the last place was the port of Amsterdam. The results indicate that the geographic location is paying a major role in the final ranking of the three ports. The competitive advantage provided by this KPF is of such extent that it will be rather difficult for the two other ports to win competition against the port of Rotterdam. However, both the port of Amsterdam and the port of Antwerp can focus on improving some KPFs connected with the level of service provided in order to start bridging the gap with the port of Rotterdam. To this extent, the sulfur cap will not distort the current competitive status quo in the ARA region. On the contrary, it will reinforce the strategic position of port of Rotterdam in the region as operators are shifting their preference toward bunkers with higher quality and at locations with higher storage and supply capacity.

This paper did not take into account potential effects of IMO’s regulation on the reduction of greenhouse gas emissions from ships nor did it take into account the potential designation of the Mediterranean Sea as an ECA. Additional studies can be carried taking into consideration (a) the effect of the designation of the Mediterranean Sea as an ECA on the bunker prices in the ARA region, (b) the ship types and sizes in order to create a more specific approach to operator’s needs, and (c) specific routes and bunkering locations in order to compare at a later stage the bunkering hub in the Northern Europe to other bunkering hubs in the world. With this research, the authors have created a starting point in answering the challenging question of the effect of sulfur cap on choosing an optimal bunkering spot and created a framework on the competitive environment of the bunkering ports in the ARA region.

## Appendix A

**Table 8:** Best performance

Port of Amsterdam	Port of Rotterdam	Port of Antwerp
	Bunker quality	Bunker price
	Bunker quantity	Availability of low sulfur bunkers
	Reliability, punctuality, and safety of bunkering services	
	Clear and precise information about services	
	Geographical advantage	
	Anchoring and docking availability	
	Simplicity/accessibility to port	
	Location of port	
	Port congestion	
	Bunkering facilities (adequacy and efficacy)	
	Navigational availability (night navigation)	
	Supply waiting time	
	Quality of bunkering services (e.g. pumping rates)	
	Availability of low sulfur bunkers	
	Usage and availability of barges

**Table 9:** Worst performance

Port of Amsterdam	Port of Rotterdam	Port of Antwerp
Bunker price		Bunker quality
Bunker quantity		Reliability, punctuality, and safety of bunkering services
Geographical advantage		Clear and precise information about services
Anchoring and docking availability		Geographical advantage
Simplicity/accessibility to port		Navigational availability (night navigation)
Location of port		Quality of bunkering services (e.g., pumping rates)
Port congestion		
Bunkering facilities (adequacy and efficacy)		
Supply waiting time		
Quality of bunkering services (e.g. pumping rates)		
Availability of low sulfur bunkers		
Usage and availability of barges		

## Appendix B

**Table 10:** Statistical analysis of the HSFO data in Rotterdam for every year since 1990.

Year	Count	Mean	Min	Max	St. Dev	Skew	Kurt
1990	52	101,74	52.00	152,00	30,863	0.269	1597
1991	52	79,01	65.50	151,00	19,806	2.689	9319
1992	52	83,44	63.00	106,50	11,741	− 0.027	2057
1993	53	66,67	54.00	83,00	8197	0.398	2055
1994	52	84,14	56.00	108,00	11,448	− 0.144	2700
1995	52	96,04	77.50	114,00	10,872	− 0.083	1412
1996	52	105,79	85.00	128,50	12,321	− 0.049	1745
1997	52	95,15	83.00	119,00	9,202	0.668	2602
1998	52	67,70	50.50	86,00	7,468	0.160	3645
1999	53	93,79	56.00	133,50	27,579	0.103	1383
2000	52	138,80	104.50	168,00	14,879	0.140	2484
2001	52	116,53	98.50	136,00	10,108	− 0.083	2208
2002	52	133,65	100.50	166,50	17,557	− 0.261	2360
2003	52	153,08	123.50	195,50	15,873	0.269	2914
2004	52	155,47	127.50	179,50	13,506	− 0.174	2113
2005	53	233,93	141.50	291,50	40,313	− 0.743	2651
2006	52	292,38	249.50	331,00	23,673	− 0.210	1880
2007	52	345,99	219.50	495,00	74,196	0.248	2218
2008	52	474,33	171.50	707,00	145,893	− 0.605	2543
2009	52	352,73	173.50	466,00	84,748	− 0.403	1714
2010	52	450,23	405.00	494,00	22,407	0.061	2129
2011	53	617,14	494.00	669,50	42,596	− 1.528	4476
2012	52	638,77	550.50	720,00	50,203	0.058	1685
2013	52	594,49	567.00	646,00	17,228	0.867	3744
2014	52	533,36	305.00	601,00	77,842	− 1.702	4712
2015	52	265,15	133.50	359,50	56,605	− 0.331	2260
2016	53	213,20	112.00	307,00	52,280	− 0.272	2120
2017	52	305,18	267.50	364,00	25,359	0.631	2647
2018	52	400,07	322.50	477,50	40,685	− 0.025	1831
2019	52	348,89	245.75	443,50	59,851	− 0.333	1672
2020	29	228,73	124.75	303,75	58,528	− 0.199	1626
ALL	1594	254,06	50.50	720,00	183,331	0.849	2485

## Footnotes

1 Transparency (corruption-free), port tariffs, duration of supply, prices of complementary services for fuel supply at berth (pilotage mooring etc.), prices of complementary services for fuel supply at anchorage, presence of restrictive environmental regulations, simplicity of crew changes, custom strictness, ship inspection thoroughness, government policies (e.g. quality control) and incentives, and bunkering rules of port.
